# Enhanced Downstream Processing for a Cell-Based Avian Influenza (H5N1) Vaccine

**DOI:** 10.3390/vaccines12020138

**Published:** 2024-01-29

**Authors:** Fang Li, Bo Liu, Yu Xiong, Zhegang Zhang, Qingmei Zhang, Ran Qiu, Feixia Peng, Xuanxuan Nian, Dongping Wu, Xuedan Li, Jing Liu, Ze Li, Hao Tu, Wenyi Wu, Yu Wang, Jiayou Zhang, Xiaoming Yang

**Affiliations:** 1 National Engineering Technology Research Center for Combined Vaccines, Wuhan 430207, China; lf1028222@163.com (F.L.); liubohust@126.com (B.L.); xiongyyyyyy@outlook.com (Y.X.); zhangzhegang@sinopharm.com (Z.Z.); 15827391276@163.com (Q.Z.); ran.qiu@outlook.com (R.Q.); pfx12706695702023@163.com (F.P.); nianxuanxuan@126.com (X.N.); 18174013525@163.com (D.W.); xuedanli@whu.edu.cn (X.L.); liujing__16@163.com (J.L.); lizewhsw@163.com (Z.L.); tuhaofirst@163.com (H.T.); wuwenyi1116@163.com (W.W.); wangyui99@163.com (Y.W.); 2 Wuhan Institute of Biological Products Co., Ltd., Wuhan 430207, China; 3 National Key Laboratory for Novel Vaccines Research, Development of Emerging Infectious Diseases, Wuhan 430207, China; 4 Hubei Provincial Vaccine Technology Innovation Center, Wuhan 430207, China; 5 China National Biotec Group Company Limited, Beijing 100029, China

**Keywords:** downstream purification process, H5N1, HPAIV, MDCK cells, Capto^TM^ Core 700, hydrophobic chromatography

## Abstract

H5N1 highly pathogenic avian influenza virus (HPAIV) infections pose a significant threat to human health, with a mortality rate of around 50%. Limited global approval of H5N1 HPAIV vaccines, excluding China, prompted the need to address safety concerns related to MDCK cell tumorigenicity. Our objective was to improve vaccine safety by minimizing residual DNA and host cell protein (HCP). We developed a downstream processing method for the cell-based H5N1 HPAIV vaccine, employing Capto^TM^ Core 700, a multimodal resin, for polishing. Hydrophobic-interaction chromatography (HIC) with polypropylene glycol as a functional group facilitated the reversible binding of virus particles for capture. Following the two-step chromatographic process, virus recovery reached 68.16%. Additionally, HCP and DNA levels were reduced to 2112.60 ng/mL and 6.4 ng/mL, respectively. Western blot, high–performance liquid chromatography (HPLC), and transmission electron microscopy (TEM) confirmed the presence of the required antigen with a spherical shape and appropriate particle size. Overall, our presented two-step downstream process demonstrates potential as an efficient and cost-effective platform technology for cell-based influenza (H5N1 HPAIV) vaccines.

## 1. Introductions

In 2023, the United States faced a severe avian influenza outbreak, marking one of the most significant incidents in the nation’s history. The H5N1 highly pathogenic avian influenza virus (HPAIV), responsible for this outbreak, led to the culling of more than 58 million poultry. The Centers for Disease Control and Prevention (CDC) reported an almost 100% mortality rate among avians infected with H5N1 highly pathogenic avian influenza virus (HPAIV), with most succumbing to the virus within 48 h [1]. From 2003 to 2018, the World Health Organization (WHO) documented 860 confirmed human cases of H5N1 HPAIV globally. Of these cases, 454 proved fatal, resulting in a death rate exceeding 50% [2]. During the 2009 H1N1 influenza pandemic, human infections with the H5N1 HPAIV were also reported, raising concerns about a potential new influenza pandemic caused by H5N1 HPAIV [3]. In contrast to seasonal influenza, characterized by mild respiratory symptoms, H5N1 HPAIV infection in humans manifests as a swiftly progressing and severe illness, leading to elevated rates of morbidity and mortality [2,4]. To address the immediate risk and potential health threats associated with H5N1 HPAIV, the development of a vaccine stands as a crucial preventive measure.

The majority of current vaccines are produced using chicken embryos, leading to a prolonged egg-based production process with uncertain yields. During avian influenza outbreaks, the availability of chicken embryos is constrained, hindering comprehensive responses. Additionally, these vaccines may contain high levels of residual ovalbumin, contributing to severe allergic reactions [5]. In contrast, cell-based production of avian influenza presents several advantages, including a strong antigenic match, potential for large-scale manufacturing, shorter production times, automated monitoring, and a reduced risk of allergic reactions [6]. Consequently, the WHO recommends shifting from the traditional use of chicken embryos to mammalian cells as a substrate for influenza virus cultures in vaccine development [7]. Mammalian cells, such as MDCK and Vero cells, are utilized for generating influenza vaccines. Among these, MDCK cells are recognized as the most advantageous cellular substrate due to their exceptional efficacy in infecting influenza viruses, rapid proliferation, and reduced susceptibility to mutation [8,9]. It is worth mentioning that the MDCK cell line represents a new cell substrate for a robust influenza vaccine production in a fully defined process [10,11,12].

The traditional process of producing influenza vaccine from chicken embryos involves clarification, ultrafiltration, and sucrose zone centrifugation [13,14]. Nevertheless, this approach falls short in removing host cell residues, specifically residual protein and residual DNA, which are crucial impurities in cell culture. In virus purification, gel filtration (GF) chromatography is widely employed to purify most viruses due to the distinct differences in molecular weight, density, and charge properties between virus molecules and impurities [15]. GF chromatography is significantly faster, more consistent, and easier to automate compared to the conventional method [16]. Presently, gel filtration chromatography using traditional Sepharose series gels is the predominant method for purifying influenza viruses. To address safety concerns associated with tumorigenesis in passaged cell lines, host cell residual DNA is effectively eliminated through nuclease digestion and ion exchange chromatography [17]. The innovative Capto^TM^ Core 700 composite packing features an inert shell without any functionality and a core with an octylamine ligand, providing molecular exclusion and ion adsorption capabilities. This design makes it an optimal choice for the removal of host cell proteins and nucleases [18,19]. Compared to conventional gel packing, this method offers several advantages, including a larger sample volume, higher flow rate, and reduced loading height. These benefits facilitate a more straightforward scaling-up process while efficiently eliminating host cell residual proteins [20]. Capto^TM^ Core700 (Cytiva, Uppsala, Sweden) was used to purify rabies virus produced in Vero cells grown in serum-free medium, and the results showed that the antigen recovery yield was 84%, the DNA and HCP concentrations were 7.25 ± 1 pg/mL and 1784.4 ± 100 ng/mL, respectively [21]. In addition, for the purification of the cell culture-derived Orf virus, a complete cellular protein removal and a host cell DNA depletion of up to 82% was possible for the steric exclusion membranes and the Capto™ Core 700 combination [22]. Hydrophobic-interaction chromatography (HIC) is commonly used as a supplementary or complementary method to anion-exchange chromatography (AEC), cation-exchange chromatography (CEC), and GF chromatography [23]. This entails chromatographic separation operations at elevated salt concentrations, employing hydrophobic resins and hydrophilic salts to control the polarity and surface tension of the mobile phase. Depending on the hydrophobic micro-regions on the molecular surfaces of the separated components, the hydrophobic residues exposed after (reversible) denaturation, or the strength of the interaction between the hydrophobic residues on the molecular surfaces exposed in high salt environments and the hydrophobic ligands of the stationary phases, the components with weakest to strongest hydrophobic interactions can be separated by using the eluent with the ionic strengths ranging from high to low in a sequential manner [24]. This enables the separation of biomolecules, relying on distinctions in the hydrophobicity of the protein molecule surface [24,25,26]. The combination of the Capto^TM^ Core 700 composite mode medium and hydrophobic interaction resin for purifying and isolating cell-based H5N1 HPAIV in vaccine development and preparation is an underexplored area in the current literature [27,28,29].

This study aimed to investigate a method for the downstream purification of a cell-based H5N1 HPAIV vaccine. The purification of H5N1 HPAIV involved the combination of the composite model medium Capto^TM^ Core 700 with HIC parameters such as sample volume, sample flow rate, column bed height, and functional group species for the two-step chromatography process using the Capto^TM^ Core 700 composite model medium and hydrophobic interaction were systematically screened and optimized. The subsequent analysis focused on evaluating residual host cell proteins and DNA, along with the hemagglutinin content of the purified samples. By detecting these elements in the purified samples, the study aimed to assess the impact of Capto^TM^ Core 700 and hydrophobic interaction two-step chromatography on the isolation and purification of H5N1 HPAIV from cellular matrices, as well as the purity of the final samples. The overarching goal of the study was to introduce a novel concept for advancing cell-based H5N1 HPAIV vaccines and lay the foundation for subsequent large-scale vaccine production.

## 2. Materials and Methods

### 2.1. Cells and Strains

The MDCK cell line was maintained by the Viral Vaccine Research and Development 2 unit at WIBP (Wuhan Institute of Biological Products). The H5N1 HPAIV vaccine strain NIBRG-14, recommended by the WHO, was procured from the National Institute of Biologics and Bioproducts Control (NIBSC), Hertfordshire, UK.

### 2.2. Virus Harvesting and Pretreatment

The H5N1 HPAIV strain was cultured in MDCK cells for 72 h in the bioreactor (MOI = 0.001). Following cultivation, the viral fluids were collected, clarified, and concentrated through ultrafiltration. The concentrated material was then stored at 4 °C for subsequent purification.

### 2.3. Capto^TM^ Core 700 Chromatography

#### 2.3.1. Bed Height

Column bed heights of 16 cm, 25 cm, and 34 cm were chosen for column loading. The linear flow rate was maintained at 100 cm/h, utilizing a phosphate buffer (PB) system comprising 150 mmol/L NaCl and 50 mmol/L PB at a pH of 7.5. Flow–through peaks from each group were individually collected. Subsequent analysis involved measuring total protein, hemagglutinin, residual protein, and residual DNA contents of the host cells. This allowed for a comparison of the impact of various column bed heights on the purification efficacy.

#### 2.3.2. Linear Flow Rates

The column bed height was set at 16 cm, and the buffer solution consisted of 150 mmol/L NaCl + 50 mmol/L PB at pH 7.5. Linear flow rates of 50, 100, and 200 cm/h were chosen. Flow–through peaks from each group were collected and analyzed to assess the impact of different linear flow rates on purification effectiveness. The comparison involved evaluating total protein content, hemagglutinin content, residual host cell protein, and residual DNA content to understand the effects of varying linear flow rates on the purification process.

#### 2.3.3. Sample Volume

The column bed height was 16 cm, with a linear flow rate of 100 cm/h, and the sampling buffer consisted of 150 mmol/L NaCl + 50 mmol/L PB at a pH of 7.5. Various bed volume (BV) ratios (25%, 50%, 100%, and 175%) were employed. Flow-through peaks from each group were collected and subjected to testing for total protein, hemagglutinin, residual host cell protein, and residual DNA content. The aim was to compare the effects of different volume ratios on the purification efficiency under constant linear flow conditions. Detailed parameter information can be found in Table 1.

### 2.4. Hydrophobic Chromatography

An appropriate volume of virus liquid, post preliminary purification using Capto^TM^ Core 700, was chosen for further purification through hydrophobic interaction chromatography. The process involved a flow rate of 100 cm/h, a pH of 7.5, and an uploading buffer of 20 mmol/LPB + 1 mol/L (NH_4_)_2_SO_4_. Subsequently, equilibration and purification were carried out using a Polar MC-HIC hydrophobic column, sequentially employing bonded phenyl and butyl groups, and regeneration steps between each use. After equilibration, the sample underwent purification using bonded phenyl and butyl, respectively, on the regenerated Polar MC-HIC hydrophobic column. Elution was performed with a gradient ranging from 1.0 to 0 mol/L (NH_4_)_2_SO_4_ over 10 bed volumes (BV). The column was then washed with water injection and 0.5 mol/L NaOH. The elution peaks were collected, and subsequent analysis involved measuring total protein content, hemagglutinin content, residual host cell protein, and residual DNA content. This comparison aimed to evaluate the purification effectiveness of hydrophobic chromatographic packing with different ligands.

### 2.5. Sodium Dodecyl Sulfate Polyacrylamide Gel Electrophoresis (SDS-PAGE) and Western Blot Analysis

Western blot analysis involves the separation of mixed protein samples via polyacrylamide gel electrophoresis (PAGE), blotting them with a special siphon or electric field device so they bind specifically to the corresponding primary antibody, which then binds to an enzyme or isotope-labelled secondary antibody, and finally, detecting the protein components specific to the target gene expression via substrate chromatography or radioautography [30]. For a more in-depth assessment of the purification efficacy at each chromatographic step, samples of purified H5N1 HPAIV-type viral fluids were subjected to separation on 8% SDS-PAGE. The separated components were subsequently transferred onto NC blotting membranes, which were then blocked with 5% BSA for 2 h at room temperature. The primary antibody, derived from standard sheep antiserum (No. 07/148 (NIBSC)), was prepared at a concentration of 1:5000 and incubated at 4 °C overnight. Following this, the secondary antibody, donkey anti-sheep IgG H&L (HRP) D110174-0100, was applied at a concentration of 1:5000 and incubated at room temperature for 2 h. The detection was carried out using a Horseradish catalase DAB color kit (Sangon Biotech, Shanghai, China).

### 2.6. HPLC Analysis

HPLC analysis, based on the principles of liquid chromatography, was employed to visually assess the separation of hemagglutinin (HA) at each chromatographic step. A suitable quantity of purified H5N1 HPAIV liquid was utilized for HPLC analysis. The separation was conducted using a Monomix MC-SEC column (300 mm × 7.8 mm, 10 µm, 1000A. Suzhou Sepax Technologies, Inc. Suzhou, China) equipped with a diode array detector (DAD). The mobile phase comprised acetonitrile-water (45:55), with a flow rate of 1.0 mL/min. The injection volume was 10 µL, and detection was performed at a wavelength of 280 nm. The column temperature was maintained at 25 °C throughout the analysis.

### 2.7. Transmission Electron Microscopy (TEM) Inspection

The characterization of purified viral samples was performed through a TEM assay using a JEM-1400 instrument from Hitachi (JEOL Ltd. Tokyo, Japan), operating at an accelerating voltage of 80 kV. Approximately 50 µL of purified H5N1 HPAIV was deposited onto a carbon-stabilized, poly (methylvinyl acetate)-coated 200-mesh copper grid. After allowing for adherence, the samples on the grid were stained with 2% phosphotungstic acid for 1 min, followed by TEM characterization to visualize and analyze the structural features of the purified virus.

### 2.8. Total Protein Content Assay

The quantification of total protein content in the purified virus products, following treatment with various chromatographic media, was conducted using the second method outlined in Part III of the General Principles of the Chinese Pharmacopoeia (2020 edition), specifically method 0731, known as the Lowry’s method.

### 2.9. Host Cell Residual DNA Detection

For extracting residual host DNA from the samples, the host cell residual DNA sample pretreatment kit (magnetic bead method) was employed. Subsequently, the residual DNA content of the samples was detected using the qPCR fluorescent probe method, which detects changes in the amount of amplified product in each cycle of PCR amplification in real time according to the fluctuation of fluorescence signal for quantitative analysis. The extraction process and DNA detection followed the instructions outlined in the MDCK Residual DNA Detection Kit (SK030209M100, Huzhou Shenke Biotechnology Co., Ltd. Huzhou, China), utilizing qPCR and a fluorescence probe for accurate measurement.

### 2.10. Host Cell Residual Protein Detection

The Folin-reagent method (Lowry method) was applied for the determination of host cell residual protein in the purified product. The phosphomolybdate-phosphotungstate in the Folin-Phenol reagent is reduced by tyrosine and tryptophan residues in proteins, producing a deep blue color (a mixture of molybdenum and tungsten orchids). Under certain conditions, the depth of the blue color is positively correlated with the amount of protein. The assessment of residual cellular protein in MDCK samples was conducted using the MDCK Cellular Residual Protein Assay Kit (F800, Cygnus, Leland, NC, USA). The specific procedures were performed in accordance with the instructions provided by the kit.

### 2.11. Hemagglutinin Content Testing

The single radial immunodiffusion (SRID) method was employed for the analysis. Standard antigen in a series of HA concentrations were performed in single radial immunodiffusion with samples together. Results were plotted as mean zone diameter versus the antigen concentration on a linear scale.

### 2.12. Statistical Analysis

All experiments were conducted in triplicate, and the data were presented as mean ± standard deviation (SD). Statistical analyses were carried out using one-way ANOVA with SPSS version 26.0 (SPSS Inc., Chicago, IL, USA). Differences were deemed statistically significant at *p* < 0.05. All data shown in the manuscript are expressed as the means ± standard deviation (SD).

## 3. Results

### 3.1. Flow Rate, Column Bed Height, and Sample Volume Affect Purification of Capto^TM^ Core 700

#### 3.1.1. The Influence of Column Bed Height on Purification Effect

**Figure 1:**
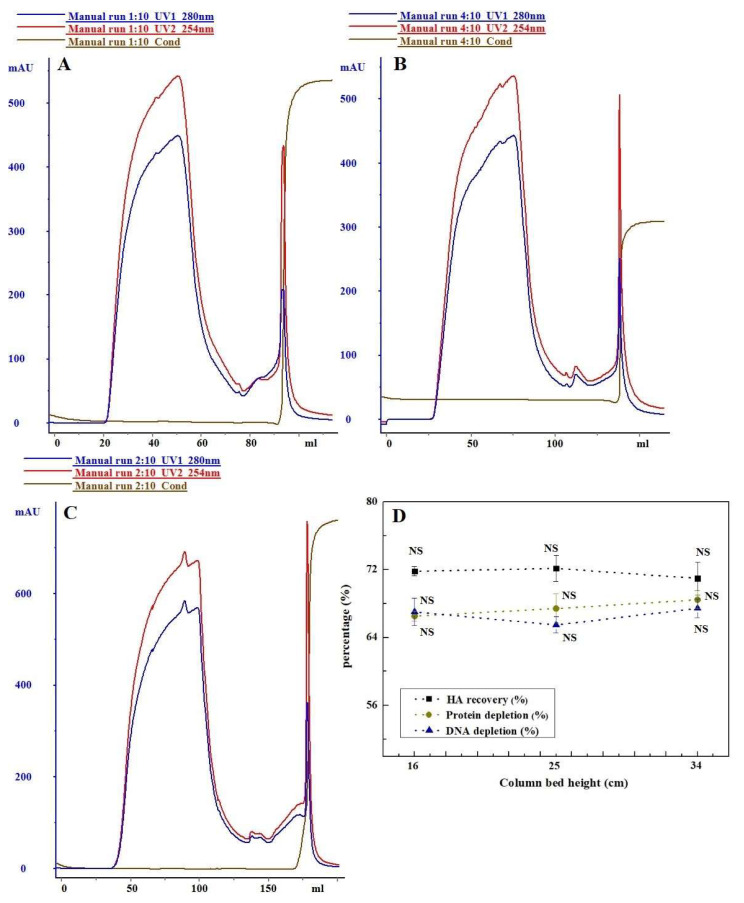
Column bed heights affected purification of Capto^TM^ Core 700: (**A**) 16 cm; (**B**) 25 cm; and (**C**) 34 cm. (**D**) Comparison of chromatographic purification effects. NS: no significance.

To assess the influence of column bed height on the purification effectiveness of H5N1 HPAIV using Capto^TM^ Core 700, parallel comparative experiments were carried out at different heights. In these experiments, two distinct protein peaks were identified at OD280. Notably, the appearance of the target peaks exhibited variability across different column bed heights. Subsequently, these peaks were isolated, captured, and subjected to analysis, with the results presented in Figure 1. Two distinct protein peaks were observed at OD280. The timing of the target peaks’ appearance varied at different column bed heights, with higher column beds resulting in later peak appearance times. There was no significant difference in the removal of impurities (total protein removal) and the recovery of haemagglutinin (HA) in the purified samples as the column bed height increased (*p* > 0.05). Additionally, the ratio of protein content to haemagglutinin concentration in the samples was 5.44, 5.58, and 5.28 for column bed heights of 16 cm, 25 cm, and 34 cm, respectively.

#### 3.1.2. The Influence of Flow Rate on Purification Effect

**Figure 2:**
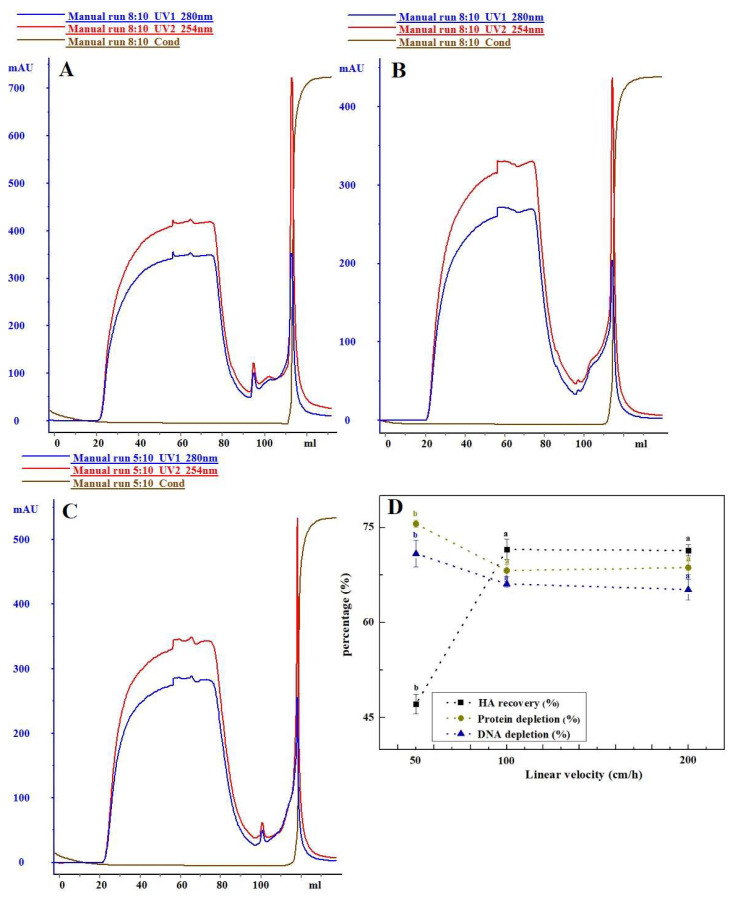
Differences in purification of Capto^TM^ Core 700 at varying linear flow rate: (**A**) 50 cm/h; (**B**) 100 cm/h; and (**C**) 200 cm/h. (**D**) Comparison of chromatographic purification effects. Data with different letters indicate significant difference at *p* < 0.05.

The distribution and binding of components in the chromatographic medium are influenced by the linear flow rate. This study analyzed the chromatographic separation of Capto^TM^ Core 700 at different flow rates (50, 100, and 200 cm/h), as illustrated in Figure 2. Increasing the linear flow rate of the loading sample from 50 cm/h to 100 cm/h resulted in a significant rise in haemagglutinin recovery from 47.13% to 71.50% (*p* < 0.05). Concurrently, there was a notable decrease in the removal of impurities (host cell residual protein, host cell residual DNA, etc.), with the total protein removal rate dropping from 75.56% to 68.19%, and the DNA removal rate decreasing from 70.86% to 66.08%. However, when the linear flow rate of the sample was further increased to 200 cm/h, there was no significant difference observed in both the recovery of haemagglutinin and the removal of impurities at the target peak (*p* > 0.05). Simultaneously, the ratio of protein to haemagglutinin concentration remained below 5:1.

#### 3.1.3. The Influence of Sample Loading Volume on Purification Effect

**Figure 3:**
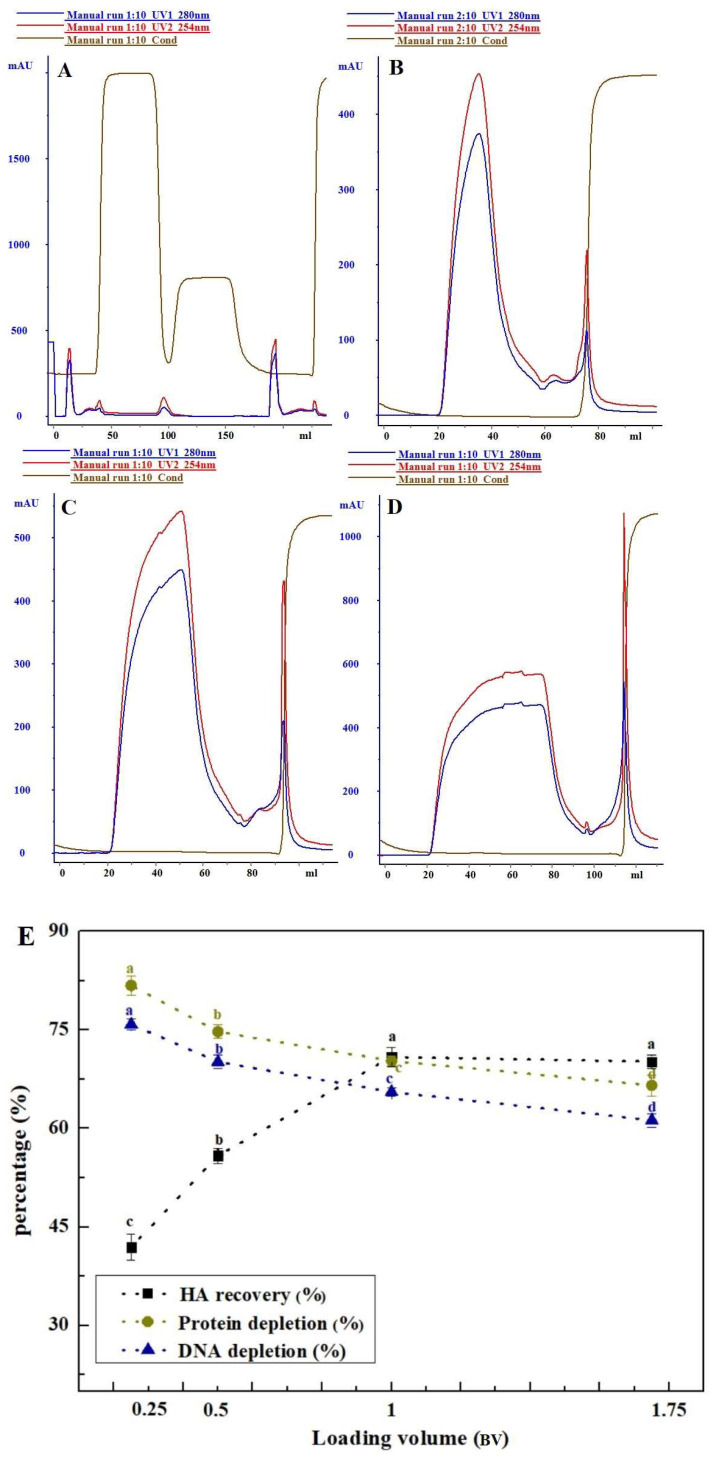
Varying sample loading volume influenced the purification of Capto^TM^ Core 700: (**A**) 0.25 BV; (**B**) 0.5 BV; (**C**) 1 BV; and (**D**) 1.75 BV. (**E**) Comparison of chromatographic purification effects. Data with different letters indicate significant difference at *p* < 0.05.

The study investigated the impact of four loading volumes (0.25, 0.5, 1, and 1.75 BV) on the chromatographic separation of Capto^TM^ Core 700, as depicted in Figure 3. With an increase in loading volume (from 0.25 BV to 1.75 BV), the impurity removal of Capto^TM^ Core 700 gradually decreased, showing a 15.23% reduction in total protein removal and a 14.60% decrease in DNA removal. However, the haemagglutinin recovery from the sample significantly increased by 29% as the sample loading volume increased from 0.25 BV to 1 BV. In comparison to the 1 BV sample volume, there was no significant difference in haemagglutinin recovery when the sample loading volume continued to increase to 1.75 BV (*p* > 0.05), with a decreasing trend in impurity removal (total protein removal: 3.74%; DNA removal: 4.33%). Table 2 presents the optimal testing parameters derived from the optimization results of Capto^TM^ Core 700. The combination of these parameters resulted in a highly successful haemagglutinin recovery rate of 72.26% for the primary purified liquid of the H5N1 HPAIV. However, the total protein removal rate was approximately 66.35%. Detailed results can be found in Table 3.

### 3.2. The Selection of Hydrophobic Chromatography Media

**Figure 4:**
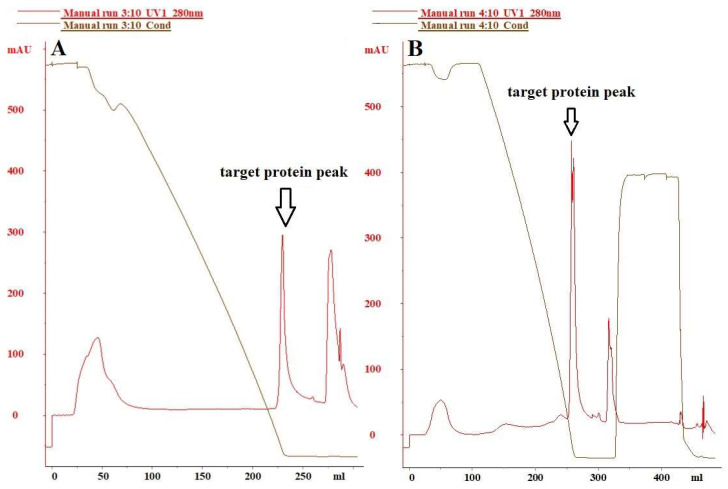
Hydrophobic interaction chromatogram of H5N1 HPAIV: (**A**) butyl; (**B**) phenyl.

The residual protein content in the host cells did not meet the required standards for vaccine products. To eliminate impurities such as residual host cell proteins and DNA in the H5N1 HPAIV purified using Capto^TM^ Core 700, an adequate amount of H5N1 HPAIV sample purified under the optimal Capto^TM^ Core 700 chromatographic conditions was subjected to additional processing using bonded butyl and bonded phenyl HIC media. The effectiveness of each purification step was assessed, with the results presented in Figure 4. The purification efficacy of hydrophobic chromatographic media with bonded phenyl groups proved significantly superior to that of hydrophobic chromatographic media with bonded butyl groups. The virus sample was loaded under buffer conditions of 20 mmol/L PB + 1 mol/L (NH_4_)_2_SO_4_, pH 7.5, and eluted with a gradient of 0–1.0 mol/L (NH_4_)_2_SO_4_ at 10 BV. Subsequently, the column was washed with water injection and 0.5 mol/L NaOH. The purification impact of the butyl-bonded hydrophobic chromatographic medium is depicted in Figure 4A, revealing a total protein removal rate of 69.21%, a host cell residual DNA removal rate of 91.26%, and an antigen recovery rate of 79.28%. Figure 4B shows the purification effect of the phenyl-bonded hydrophobic chromatographic medium, where hemagglutinin and impurities were effectively separated through gradient elution with 0–1.0 mol/L (NH_4_)_2_SO_4_ concentration. The total protein removal rate reached 76.27%, significantly surpassing that of the hydrophobic chromatographic medium with bonded butyl groups. Host cell residual protein and host cell residual DNA were reduced to (2112.60 ng/mL, 6.4 ng/mL), respectively. Additionally, the antigen recovery reached 87.14% (68.59 µg/mL).

### 3.3. The Characterization, Purity, and Morphological Analysis of Purified Virus

#### 3.3.1. SDS-PAGE and Western Blot Analysis

**Figure 5:**
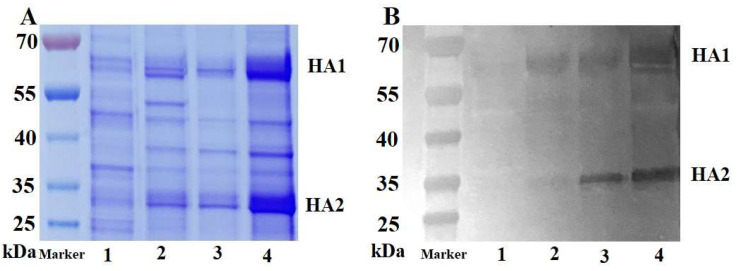
SDS-PAGE (**A**) and Western blot (**B**) analyses conducted on the H5N1 HPAIV. Band 1 represents the clarified viral suspension, band 2 signifies the virus ultrafiltration concentrate, band 3 corresponds to the virus purified by Capto^TM^ Core 700, and band 4 indicates the purified product after the second purification step.

The purification process involved four distinct samples: clarified viral suspension, virus ultrafiltration concentrate, virus purified by Capto^TM^ Core 700, and the purified product after the second purification step (Capto^TM^ Core 700 combined with hydrophobic chromatography). These samples were labeled as samples 1, 2, 3, and 4. All samples underwent reduced SDS-PAGE and were subjected to analysis through Coomassie Brilliant Blue staining to compare the content of HA protein. Additionally, the purification process was validated using a sample reduction Western blot to confirm the specificity of the target protein, as illustrated in Figure 5. In the Western blot experiment, the primary antibody used was standard sheep anti-HA serum (concentration of 1:5000), and the secondary antibody used was donkey anti-sheep IgG H&L (HRP) (concentration of 1:5000). Specific bands, representing the target proteins HA1 and HA2 were observed in all virus sample groups, between 55~70 kDa and 25~35 kDa, respectively (Figure 5B). SDS-PAGE results indicated that the HA protein content in sample 4 was significantly higher than that in the other three groups, following the combined hydrophobicity using hydrophobic chromatography and Capto^TM^ Core 700 composite mode chromatography, while keeping the sample volumes equal.

#### 3.3.2. HPLC Analysis

**Figure 6:**
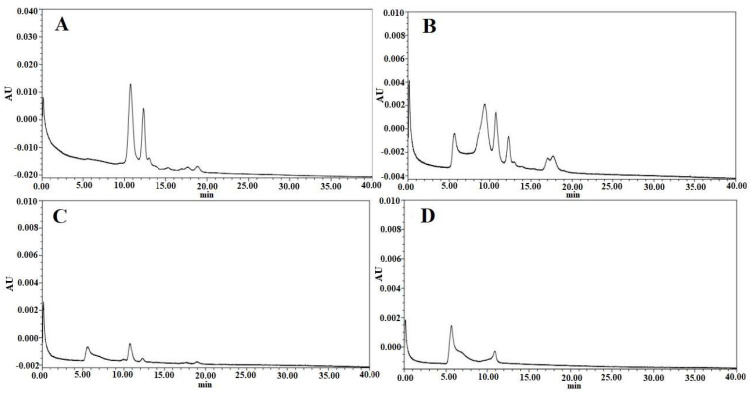
High−performance liquid chromatograms of samples from the H5N1 HPAIV purification process with each peak corresponding to different stages of the process: (**A**) clarified viral suspension, (**B**) virus ultrafiltration concentrate, (**C**) virus purified by Capto^TM^ Core 700, and (**D**) purified product after the second purification step.

The purity of the HA protein in samples 1, 2, 3, and 4 was assessed using HPLC analysis. The corresponding data are depicted in Figure 6A, Figure 6B, Figure 6C, and Figure 6D, respectively. The target protein peak began to emerge 5–10 min after the ultrafiltration concentration treatment of the clarified viral suspension. Subsequently, further preliminary purification of the ultrafiltration concentrate through Capto^TM^ Core 700 notably reduced the number of impurity peaks. The peak value of the target protein peak was slightly lower, attributed to the increase in the collected sample volume after Capto^TM^ Core 700 gel filtration chromatography compared to the loading sample. Ultimately, following purification via a phenyl-bonded hydrophobic chromatography medium, the peak value of the target protein in the viral fluid sample increased, and the number of impurity protein peaks was further reduced.

#### 3.3.3. TEM Analysis

**Figure 7:**
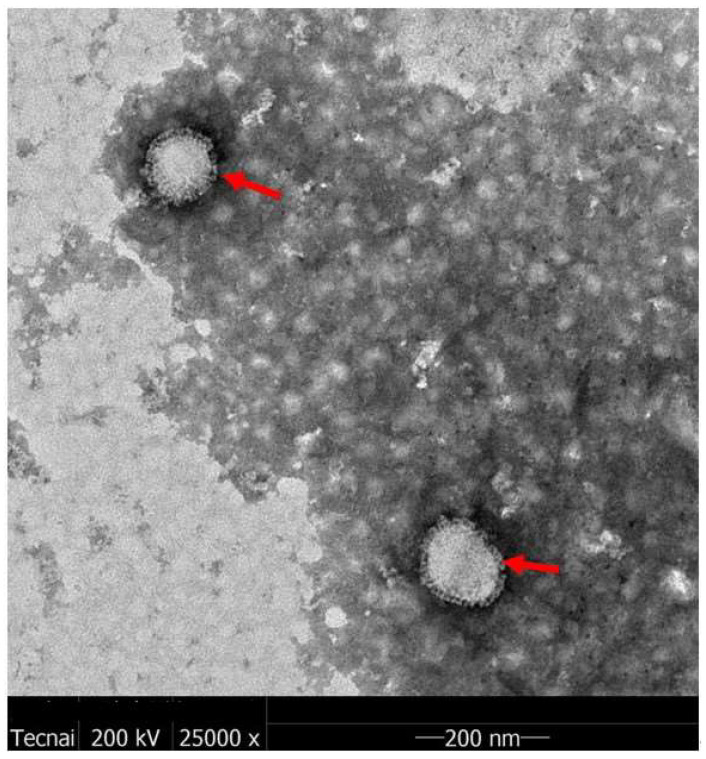
Transmission electron microscopy patterns of influenza H5N1 HPAIV (The H5N1 particles highlighted by the red arrow).

The H5N1 HPAIV sample underwent a two-step purification process before being subjected to TEM analysis. Figure 7 presents the results of this analysis, illustrating the virus particles in the purified liquid. The H5N1 HPAIV particles exhibit a solid spherical shape with a diameter of approximately 100 nm (highlighted by the red arrow). A vesicle membrane envelops the virus surface, adorned with numerous spines representing the HA and neuraminidase (NA). The virus’s structure aligns with the typical characteristics of influenza viruses and closely resembles H5N1 HPAIV reported in existing literature [31].

## 4. Discussion

Vaccines, designed for healthy populations, prioritize safety. The U.S. Food and Drug Administration (FDA) notes potential carcinogenicity for DNA gene fragments exceeding 200 bp (www.fda.com, Microsoft Edge, accessed on 5 July 2023). The European Pharmacopoeia stipulates a maximum residual DNA limit of 10 ng/dose [32,33]. In the manufacturing process involving MDCK cells, mitigating the tumorigenic risk associated with host cell residual DNA is commonly achieved through nuclease digestion and ion exchange chromatography. Capto^TM^ Core 700, a recently developed composite filler, features an unfunctionalized inert shell and an octylamine ligand in its core. This dual functionality in molecular exclusion and ion adsorption makes it effective in eliminating HCP and nucleases [34,35]. Capto^TM^ Core 700 has proven its utility in producing vaccines against diverse viruses, including rotavirus, rabies, influenza, and encephalitis B. It has also been applied in generating transgenic virus-like particles [36,37,38,39,40]. Presently, there is insufficient information regarding the efficacy of integrating Core 700 and HIC in purifying the H5N1 HPAIV vaccine. This study seeks to investigate a downstream purification method for the cell–based H5N1 HPAIV vaccine. A Capto^TM^ Core 700 composite mode medium, in conjunction with HIC, was employed to refine the avian influenza strain H5N1 HPAIV. The focus was on evaluating the combined outcomes of the two chromatographic techniques for refining the H5N1 HPAIV strain. After a two-step purification process, HA was successfully recovered at a rate of 68.16%. Furthermore, the levels of HCP and DNA were reduced to 2112.60 ng/mL and 6.4 ng/mL, respectively, meeting the residual DNA requirement of 10 ng/agent set by the European Pharmacopoeia [33]. However, the purification of cell culture-derived influenza A virus via continuous anion exchange chromatography on monoliths allowed the depletion of >98% of the DNA and >52% of the total protein [41].

The investigation initially explored the effects of different experimental factors, such as column loading height, linear flow rate, and sample volume, on sample purification using Capto^TM^ Core 700. Preliminary purification of samples using varied column bed heights revealed no significant differences in the recoveries of haemagglutinin and the removal of impurities like host cell residual protein and host cell residual DNA (*p* > 0.05). Capto^TM^ Core 700, a new composite packing material, consists of an inert, unfunctionalized shell and a core with an octylamine ligand. The core performs dual functions—molecular exclusion and ion adsorption—critical for efficient HCP removal. Unlike traditional molecular sieve gel media, Core 700 achieves separation without requiring a high column bed height. Increasing the linear flow rate from 50 cm/h to 100 cm/h reduced the retention time of the viral sample in the column, resulting in a substantial increase in haemagglutinin retrieval to 71.50% (*p* < 0.05). However, this also led to a notable decrease in contaminant elimination. Lower up-sampling flow rates extended sample retention in the column bed, allowing some virus particles to pass through the inert shell via a precisely sized notch. This led to sample diffusion and zone broadening. Tania P. Pato (2019) conducted a study on a yellow fever virus vaccine and demonstrated a 41.6% increase in antigen recovery by enhancing the up-sampling linear flow rate from 200 cm/h to 500 cm/h, with a corresponding spike in HCP content [19]. The reduction in the removal of residual DNA from host cells is attributed to the DNA fragment size exceeding the bead pore size (with a 700 kDa molecular weight threshold) [42]. This results in shorter retention times hindering DNA fragments from promptly binding to the octylamine ligand. Furthermore, this study established an inverse relationship between Core 700′s ability to eliminate impurities and the volume of sample uptake. Compared to a sample volume of 0.25 BV, total protein and residual DNA removal decreased, while haemagglutinin recovery increased at a sample volume of 1 BV, causing a shift in the total protein to haemagglutinin content ratio. The dynamic loading for Capto^TM^ Core 700, according to the manufacturer, is 13 mg of ovalbumin per mL of packing at a flow rate of 200 cm/h [33]. Higher protein loadings increase the importance of up-sample volume relative to the column volume, affecting separation and purification results due to pore diffusion effects. Higher viral loadings may negatively impact resolution [21]. Optimal test parameters were chosen based on Capto^TM^ Core 700 chromatography test conditions. After the combined purification process, the final haemagglutinin recovery rate in the primary purification solution of H5N1 HPAIV reached 72.26%, slightly exceeding the antigen recovery of chicken embryo stromal influenza virus purified via Core 700 by Hans Blom (69%) [37]. Similarly, the total virus yield for cell culture-derived influenza A/PR/8/34 (H1/N1) virus of a membrane-based purification process was 75% [43].

To further eliminate remaining proteins (3145 ± 116.05 ng/mL) from the host cells of the primary H5N1 HPAIV purification solution, we chose Polar MC-HIC, using the hydrophobic nature of phenyl over butyl [44]. This choice facilitated the ongoing isolation and purification of the viral fluid previously processed by Capto^TM^ Core 700. Additionally, the presence of 1 mol/L (NH_4_)_2_SO_4_ enhanced the binding of target proteins to the hydrophobic medium, increasing their conformational stability while decreasing solubility. Consequently, Polar MC-HIC, equipped with phenyl functional groups, was selected for the subsequent isolation and purification of the viral fluid treated with Capto^TM^ Core 700. The tight binding of target proteins to the hydrophobic medium induced by 1 mol/L (NH_4_)_2_SO_4_ resulted in increased conformational stability and reduced solubility. Employing concentration gradient elution with (NH_4_)_2_SO_4_ led to improved antigen separation from heteroproteins, accompanied by a decrease in residual proteins and host cell DNA to (2112.60 ng/mL, 6.4 ng/mL), respectively. Furthermore, the antigen’s recovery rate reached 87.14% (68.59 µg/mL). In a similar manner, Influenza A viral fluids were isolated by Thomas Weigel through initial purification with Capto Q by HIC (with polypropylene glycol as a functional group), achieving the removal of 57.3% total protein and 45.7% host cell residual DNA [45]. Finally, to validate the separation of H5N1 HPAIV samples and characterize their purity, SDS-PAGE, Western blot, HPLC, and TEM were employed. The results demonstrated that after applying Capto^TM^ Core 700 and hydrophobic interaction chromatography in the two-step purification process, the H5N1 HPAIV samples exhibited significant purity improvement, with a noteworthy removal of impurities.

The Con A agarose medium chromatography resin is efficient in purifying glycoproteins containing mannitol and glucose residues, along with other sugar types. Moreover, affinity chromatography resin proves advantageous for the purification of glycoproteins and glycolipids, offering high specificity and efficient recovery of target proteins [46]. In addition, alternative ion chromatography has been utilized for the analysis and purification of the influenza virus HA glycoprotein.

Numerous studies highlight the challenge of removing host cell residual proteins and DNA from cell-based vaccines using the traditional downstream purification approach applied to chicken embryo-based vaccines [47,48]. This often necessitates multiple costly and labor-intensive downstream purification steps. Further investigation is warranted, considering the possibility that the binding of DNA fragments to viral particles and viral protein aggregates during the production process might be a contributing factor [49].

The results of this study demonstrate that the composite model medium Capto^TM^ Core 700, coupled with HIC, provides a straightforward and highly effective method for purifying H5N1 HPAIV. In addition, the reproducibility and reliability of the downstream purification process was validated in the production of the clinical samples of H5N1 HPAIV vaccine. Although only one type of strain is used for the development of the two-step purification process, a specific focus also was laid on the investigation of the downstream purification process of H7N9, it was found that the two-step purification process was suitable for H7N9 as well and achieved ideal purification effect. In summary, this purification method is pivotal in laying the foundation for the subsequent scale-up of the downstream process for cell culture-derived influenza vaccine production.

## Figures and Tables

**Table 1:** Parametric variables (column bed height, linear flow rate, and sample volume) were adjusted to assess their effect on the purification of Capto^TM^ Core 700.

Parameters	Variables
Bed Heights (cm)	16
	25
	34
Linear flow rates (cm/h)	50
	100
	200
Sample volume (%BV)	25
	50
	100
	175

**Table 2:** Optimization steps for Capto^TM^ Core 700 chromatography.

Step	Parameter
System	ÄKTA™ purifier (Cytiva, Uppsala, Sweden)
Column	XK16/40 (Cytiva, Uppsala, Sweden), column height = 16 cm, Capto^TM^ Core 700
Sample	H5N1 HPAIV (NIBSC, Hertfordshire, UK) ultrafiltration concentrate
Sample load	100% BV, 100 cm/h
Running buffer	150 mmol/L NaCl + 20 mmol/L PB, pH 7.5, 100 cm/h
Wash	150 mmol/L NaCl + 20 mmol/L PB, pH 7.5, 100 cm/h
Cleaning in place (CIP)	0.5 M NaOH, 60 cm/h

**Table 3:** Test results of Capto^TM^ Core 700 under optimal chromatographic conditions.

Indicator	Value
HA Recovery (%)	72.26 ± 2.11
Protein depletion (%)	66.35 ± 1.95
DNA depletion (%)	65.16 ± 2.18
Total protein concentration/HA concentration	4.56 ± 0.76
HA (µg/mL)	45.86 ± 4.29
DNA (ng/mL)	83 ± 6.86
HCP (ng/mL)	3145 ± 116.05

## Data Availability

All data supporting the findings of this study are available within the manuscript. Any additional data are available from the corresponding author upon reasonable request.
